# Regeneration of Cochlear Synapses by Systemic Administration of a Bisphosphonate

**DOI:** 10.3389/fnmol.2020.00087

**Published:** 2020-07-14

**Authors:** Richard Seist, Mingjie Tong, Lukas D. Landegger, Sasa Vasilijic, Hiroshi Hyakusoku, Sachiyo Katsumi, Charles E. McKenna, Albert S. B. Edge, Konstantina M. Stankovic

**Affiliations:** ^1^Eaton-Peabody Laboratories, Department of Otolaryngology – Head and Neck Surgery, Massachusetts Eye and Ear, Boston, MA, United States; ^2^Department of Otolaryngology – Head and Neck Surgery, Harvard Medical School, Boston, MA, United States; ^3^Department of Otorhinolaryngology-Head and Neck Surgery, Paracelsus Medical University, Salzburg, Austria; ^4^Department of Otorhinolaryngology-Head and Neck Surgery, Medical University of Vienna, Vienna, Austria; ^5^Department of Otorhinolaryngology, Yokosuka Kyosai Hospital, Kanagawa, Japan; ^6^Department of Chemistry, University of Southern California, Los Angeles, CA, United States; ^7^Speech and Hearing Bioscience and Technology Program, Harvard Medical School, Boston, MA, United States; ^8^Harvard Stem Cell Institute, Cambridge, MA, United States; ^9^Program in Therapeutic Science, Harvard Medical School, Boston, MA, United States

**Keywords:** auditory nerve, cochlear synaptopathy, noise-induced hearing loss, hidden hearing loss, bisphosphonate, synapse regeneration, mevalonate pathway, drug repurposing

## Abstract

Sensorineural hearing loss (SNHL) caused by noise exposure and attendant loss of glutamatergic synapses between cochlear spiral ganglion neurons (SGNs) and hair cells is the most common sensory deficit worldwide. We show here that systemic administration of a bisphosphonate to mice 24 h after synaptopathic noise exposure regenerated synapses between inner hair cells and SGNs and restored cochlear function. We further demonstrate that this effect is mediated by inhibition of the mevalonate pathway. These results are highly significant because they suggest that bisphosphonates could reverse cochlear synaptopathy for the treatment of SNHL.

## Introduction

Disabling hearing loss affects 466 million people worldwide and is expected to increase to 900 million by 2050 (Wilson et al., 2017). The annual cost of unaddressed hearing loss is $750 billion globally (Wilson et al., 2017). Most of this burden is due to sensorineural hearing loss (SNHL), which originates from defects in the cochlea, the tiny organ nestled within the densest bone in the body. Despite these statistics, pharmacological therapies for SNHL are virtually non-existent. Glucocorticoids are the only drug class that has shown some efficacy for idiopathic sudden SNHL, which is a small minority of SNHL. Yet, the utility of this treatment remains unclear because randomized controlled trials have reported contradictory outcomes and have typically relied on a small number of patients (Crane et al., 2015).

Frequent causes of SNHL include exposure to loud noise and aging, which damages cochlear sensory hair cells, resulting in elevated thresholds on the clinical audiogram. Sound-induced vibrations stimulate cochlear sensory hair cells, neurotransmitter release, excitation of the auditory nerve, and transmission of the neural impulses to the auditory cortex. However, recent studies in animals suggest that well before this overt hearing loss is measurable, a more insidious but likely more common process takes place, causing permanent loss of synapses between inner hair cells (IHCs) and subsets of cochlear nerve fibers (Kujawa and Liberman, 2009; Jensen et al., 2015; Liberman et al., 2016; Liberman and Kujawa, 2017). This cochlear synaptopathy alters auditory information processing and likely contributes to compromised word recognition ability, difficulties understanding speech in noise, tinnitus, and hyperacusis (Bauer et al., 2007; Schaette and McAlpine, 2011; Rüttiger et al., 2013; Liberman and Kujawa, 2017).

We previously showed that mice deficient in osteoprotegerin (OPG), a key regulator of bone remodeling, developed SNHL characterized by degeneration of the cochlear nerve (Kao et al., 2013). In wild-type mice, OPG was secreted by cells of the spiral ganglion, and OPG deficiency activated extracellular signal-regulated kinase (ERK), sensitized cells of the spiral ganglion to apoptosis, and inhibited proliferation and survival of cochlear progenitor cells. These phenotypes were rescued *in vitro* by treatment with exogenous OPG, an ERK inhibitor, or a bisphosphonate, zoledronate (Kao et al., 2013). This finding suggested that pathways involved in bone homeostasis were also important for cochlear sensorineural integrity. Moreover, these findings in mice supported the effect of zoledronate, which is approved for the treatment of osteoporosis and related bone diseases (Pazianas and Abrahamsen, 2011), as an off-label treatment in otosclerosis, a disease characterized by pathologic remodeling of the cochlear bone (Quesnel et al., 2012). Because word recognition is a sensitive metric of cochlear neural function, a surprising clinical observation (Quesnel et al., 2012; Jan et al., 2017) that bisphosphonates could stabilize SNHL and improve word recognition scores in patients with otosclerosis, together with our previous results on the influence of the drug on cochlear sensory cells (Kao et al., 2013), prompted us to assess the effects of bisphosphonates on cochlear synaptopathy.

## Results

**FIGURE 1:**
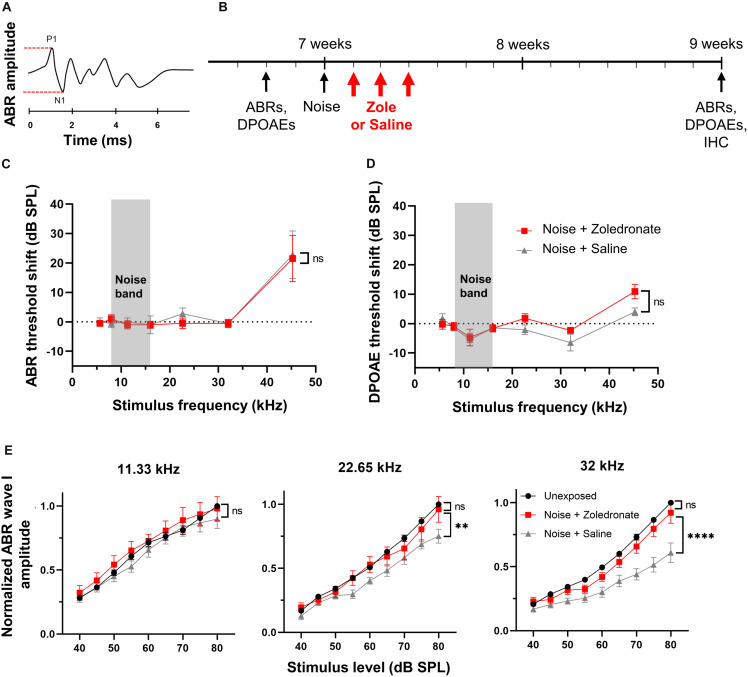
Zoledronate treatment rescues wave I ABR amplitude after neuropathic noise exposure in mice. **(A)** Schematic of ABR waveform. ABR wave I amplitude is measured between P1 and N1, as indicated by dashed lines. **(B)** Schematic timeline of the experimental protocol in mice. Animal age is specified in weeks. **(C)** Mean ABR threshold shifts and **(D)** mean DPOAE threshold shifts at 2 weeks post noise exposure relative to 2–4 days pre-exposure. Systemic administration of zoledronate did not affect ABR and DPOAE thresholds compared to control animals receiving the same volume of systemically administered vehicle (saline). Threshold shifts for each individual animal were used to calculate the means. Data are presented as means ± SEM. ns: not significant. *N* = 10 mice for Noise + Zoledronate group; *N* = 9 mice for Noise + Saline group. The gray rectangle indicates noise band. **(E)** (Left) Noise exposure did not affect ABR wave I thresholds at 11.33 kHz, as expected for the noise parameters we used. (Middle) While the noise-induced reduction in ABR wave I amplitude at 22.65 kHz was statistically significant (^∗∗^*P* = 0.0023), our criterion for statistical significance was only met at the highest tested SPL, 80 dB; this reduction was reversed with zoledronate. (Right) Systemic zoledronate administration 1, 2, and 3 days after exposure to 8–16 kHz noise at 97 dB SPL for 2 h led to near-complete recovery of ABR wave I amplitude at 32 kHz (right)—the frequency region of maximal neuropathic damage. ABR wave I amplitudes in zoledronate-treated mice were statistically indistinguishable from unexposed mice. Control, vehicle-treated animals demonstrated a statistically significant noise-induced reduction in ABR wave I amplitude of ∼40% (^****^P < 0.0001). ABR wave I amplitudes were normalized to the amplitude of ABR wave I at 80 dB in the same animal prior to noise exposure. Data are presented as means ± SEM. *N* = 10 mice for Noise + Zoledronate group; *N* = 9 mice for Noise + Saline group.

Loss of cochlear synapses was assessed morphologically and physiologically. Morphologically, afferent synapses at the base of IHCs were counted. Physiologically, wave I of the auditory brainstem response (ABR) was measured. This wave reflects the summed electrical signal from the first-order neurons of the auditory system, the spiral ganglion neurons (SGNs), which transmit the signal elicited by sound in the mechanotransducer cells, the hair cells, to the brainstem and then higher central auditory pathways. Wave I amplitude (red lines) is calculated by adding absolute values of positive deflection (P1) and negative deflection (N1) (Figure 1A). A reduction in wave I amplitude, without permanent alteration in the sound level required to elicit the response, and without loss of hair cells, as detected by the distortion product otoacoustic emissions (DPOAE), is the electrophysiological hallmark of cochlear synaptopathy (Kujawa and Liberman, 2006). Prior work by us (Jensen et al., 2015) and others (Kujawa and Liberman, 2009; Suzuki et al., 2016) defined noise parameters that cause neuropathic hearing loss in the basal half of the murine cochlea.

Here, we used this type of neuropathic noise exposure to evaluate whether systemic administration of zoledronate after noise exposure could reverse cochlear synaptopathy *in vivo* (Figure 1B). We elicited cochlear synaptopathy by exposure of mice to noise. Consistent with previous reports, ABR (Figure 1C) and DPOAE (Figure 1D) thresholds were unaffected 2 weeks after exposure to 8–16 kHz octave-band noise at 97 dB SPL for 2 h, with the exception of small threshold elevations at 45 kHz, despite significant elevation 6 and 24 h (Kujawa and Liberman, 2009; Jensen et al., 2015) after noise exposure. ABR thresholds are not permanently affected because IHC-SGN synapses remaining after neuropathic noise exposure have low thresholds and specialize in detecting softest sounds; in contrast, IHC-SGN synapses eliminated by neuropathic noise exposure have high thresholds (Furman et al., 2013). DPOAE thresholds are not permanently affected because outer hair cells are not damaged at this level of noise exposure.

To assess the effect of zoledronate on cochlear synaptopathy, we measured amplitudes of ABR wave I (Figure 1E). Consistent with previous reports (Kujawa and Liberman, 2009; Suzuki et al., 2016), we confirmed significant reductions in ABR wave I amplitude in the 32-kHz region of the cochlea 2 weeks after noise exposure. Noise-exposed animals received zoledronate (*N* = 10 animals) or carrier (*N* = 9 animals) subcutaneously, 1, 2, and 3 days after noise exposure. The zoledronate-treated group showed a statistically significant increase of 51% in ABR wave I amplitude 2 weeks after noise exposure at multiple sound pressure levels: 80 dB (*P* < 0.0001), 75 dB (*P* < 0.0001), 70 dB (*P* = 0.0001), and 65 dB (*P* = 0.0132). The ABR wave I amplitude in the zoledronate-treated group was statistically indistinguishable from the pre-noise level.

While noise exposure caused smaller reductions in ABR wave I amplitude at 22.65 kHz than at 32.00 kHz, the reduction was completely reversed by zoledronate treatment. In contrast, the noise exposure did not affect ABR wave I amplitudes at 11.33 kHz (Figure 1E), consistent with published studies (Kujawa and Liberman, 2009; Jensen et al., 2015), and zoledronate had no effect in this frequency region.

**FIGURE 2:**
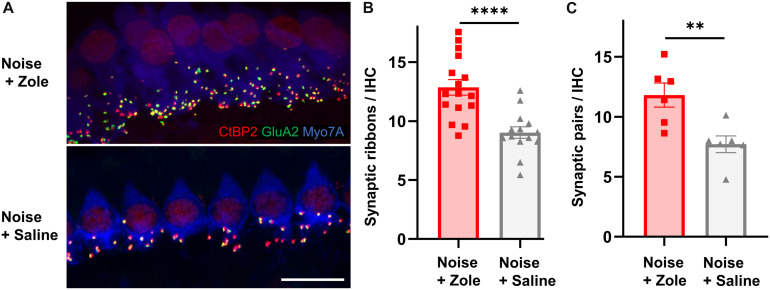
Zoledronate treatment regenerates afferent synapses on IHCs after neuropathic noise exposure in mice. **(A)** Representative cochlear whole mounts illustrating maximal projections of the IHC area in the 32-kHz region immunostained for CtBP2 (red), GluA2 (green), and Myo7A (blue) revealed the synapses between auditory nerve terminals and IHCs in mice exposed to 97 dB SPL octave band white noise (8–16 kHz) for 2 h followed by subcutaneous injection with zoledronate (“Zole,” top) or saline (bottom) 1, 2, and 3 days after noise exposure, and sacrificed 2 weeks after noise exposure. Scale bar: 10 μm. **(B)** CtBP2-positive IHC synaptic ribbon counts 2 weeks after noise exposure revealed significantly larger counts at 32 kHz in animals receiving zoledronate than in control animals receiving saline. ^****^P < 0.0001. Data are presented as means ± SEM. *N* = 16 ears for Noise + Zoledronate group; *N* = 14 ears for Noise + Vehicle group. **(C)** Synaptic counts of CtBP2-positive pre-synaptic ribbons and juxtaposed GluA2-positive post-synaptic densities at 32 kHz confirmed significantly more synapses in animals receiving zoledronate than in control animals receiving saline. ^∗∗^*P* < 0.01. Data are presented as means ± SEM. *N* = 6 ears for each group.

Because noise-induced cochlear neuropathy can be caused by synaptic loss, we analyzed synapses between IHCs and peripheral axons of SGNs (Kujawa and Liberman, 2009; Jensen et al., 2015). Ninety-five percent of SGNs are bipolar, myelinated neurons that form ribbon synapses with IHCs (Nayagam et al., 2011). Each of these synapses consists of a pre-synaptic CtBP2-positive ribbon in the basolateral surface of IHCs and a juxtaposed post-synaptic GluA2-positive density in the SGN. Compared to IHC synaptic counts in noise-exposed animals that received vehicle, the number of synapses in the zoledronate-treated ears was increased (Figure 2A; immunostaining for CtBP2 and GluA2 in the 32 kHz region). The synaptic recovery is quantified in Figure 2B and shows a dramatic 42% increase in synaptic ribbons (*P* < 0.0001) as well as a 55% increase in CtBP2- and GluA2-positive synaptic pairs (*P* < 0.01) after zoledronate treatment. Notably, the zoledronate-induced increase in synaptic counts in the 32-kHz region corresponded to the increase in suprathreshold ABR wave I amplitude (Figure 1E), thereby providing structural evidence for the observed functional outcome.

**FIGURE 3:**
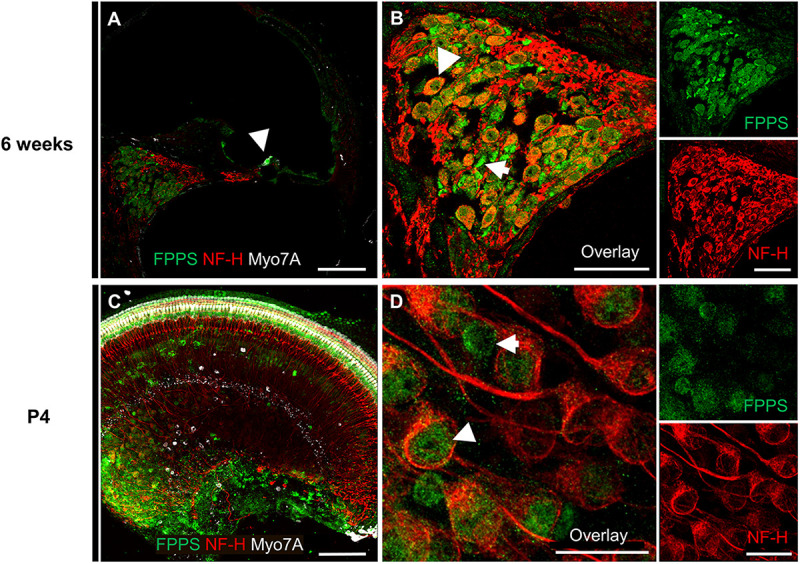
Immunolocalization of FPPS in the murine cochlea. **(A)** Low-magnification cross-section through the cochlea at 6 weeks revealed strong FPPS immunoreactivity (green) in the Myo7A-positive area of hair cells (white, arrow) and NF-H-positive SGNs (red). **(B)** High-magnification cross-section of the spiral ganglion co-immunostained for SGNs with NF-H (red) and FPPS (green) shows a weak positive signal in SGNs (white arrowhead), and a strong positive signal adjacent to SGNs where spiral ganglion Schwann or satellite glial cells reside (white arrow). **(C)** Low-magnification view of a neonatal cochlear explant at P4 reveals a strong FPPS signal (green) in the area of hair cells and the spiral ganglion. **(D)** High magnification of the spiral ganglion shows FPPS immunostaining (green) in (white arrowhead) and adjacent to (white arrow) SGNs (red). Scale bars: a, c, 100 μm; b, 50 μm; d, 20 μm. Representative images from *N* = 3 6-week-old mice and *N* = 3 4-day-old mice.

To further investigate the molecular effects of zoledronate, we examined the expression of farnesyl pyrophosphate synthase (FPPS), the main target of zoledronate (Kavanagh et al., 2006). The enzyme was localized to the spiral ganglion and IHC region of both mature and neonatal mice by fluorescence immunohistochemistry (Figure 3). In the mature cochlea, co-immunostaining for FPPS and neurofilament H (NF-H) to label SGNs revealed FPPS expression in SGNs and in NF-H-negative cells adjacent to SGNs (Figures 3A,B). In the 4-day-old cochlea, FPPS was highly expressed in the organ of Corti and SGN region (Figures 3C,D).

**FIGURE 4:**
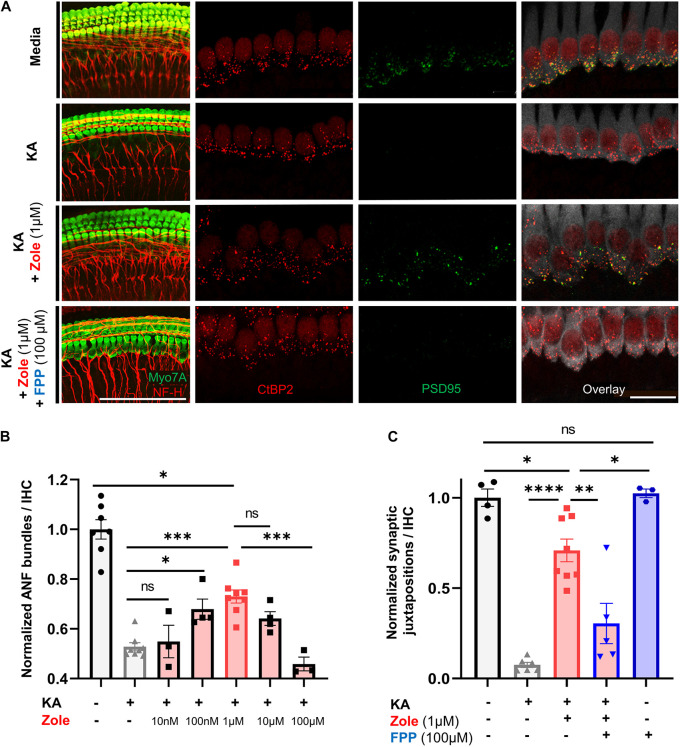
Zoledronic acid regenerates IHC-SGN synapses by inhibition of the mevalonate pathway. **(A)** Representative images of cochlear explants in culture media alone (first row), treated with 0.5 mM KA to induce synaptopathic damage (second row), co-treated with KA and zoledronate (third row), or co-treated with KA, zoledronate, and 100 μM FPP (fourth row). First column: immunostaining for Myo7A-positive hair cells and NF-H-positive NF bundles (red). Second–fourth column: CtBP2-positive presynaptic ribbons (red) in Myo7A-positive hair cells (white) and PSD95-positive post-synaptic densities (green) in juxtaposed SGN peripheral axons that are affected by experimental manipulation. Scale bar: 100 μm (first column) and 20 μm (second–fourth column). **(B)** Quantification of NF bundles per hair cell in cochlear explants normalized to media-only treatment. Data are presented as means ± SEM per group. *N* = 3–8 explants per group. ns: not significant, ^∗^*P* < 0.05, ^∗∗^*P* < 0.01, ^∗∗∗^*P* < 0.001. **(C)** Quantification of CtBP2 and PSD95 juxtapositions per hair cell in cochlear explants normalized to media-only treatment. Data are presented as means ± SEM. *N* = 3–8 explants per group. ns: not significant, ^∗^*P* < 0.05, ^∗∗^*P* < 0.01, ^∗∗∗^*P* < 0.001, ^****^P < 0.0001.

The postulated mechanism for disruption of synapses by noise involves excessive release of glutamate into the synaptic cleft with attendant damage to the postsynaptic neurons (Liberman and Kujawa, 2017). We used an *in vitro* model of excitatory cochlear synaptopathy that we have employed previously (Wang and Green, 2011; Kempfle et al., 2018; Yamahara et al., 2019) to further probe the mechanism of the regenerative effect of the drug on cochlear synapses. In this model, cochlear explants consisting of hair cells and attached SGNs are exposed to kainic acid (KA), a selective ionotropic glutamate receptor agonist. Compared to control explants without KA treatment, explants cultured for 2 h with 0.5 mM KA and assessed 24 h later showed a severe reduction in the number of neurofilament (NF) bundles per IHC. Co-incubation with zoledronate showed a dose-dependent recovery, with the highest observed response at 1 μM. Higher doses of zoledronate (i.e., 10 and 100 μM) were neurotoxic, so we focused on 1 μM zoledronate for further experiments (Figures 4A,C). As the IHC synapse is not yet mature in these neonatal tissues, we refer to pre- and postsynaptic juxtapositions, as defined by CtBP2-expressing synaptic ribbons and PSD95-positive post-synaptic densities (Figure 4C). Relative to control explants without KA treatment (Figure 4B), explants cultured for 2 h with 0.5 mM KA and assessed 24 h later showed a severe reduction in the number of juxtapositions. Incubating explants with 1 μM zoledronate in culture medium for 24 h after 2 h of KA damage showed regeneration of synapses. To test whether this effect of zoledronate was caused by inhibition of FPPS, we co-incubated explants with zoledronate and its direct downstream intermediate in the mevalonate pathway, farnesyl pyrophosphate (FPP). The effect of zoledronate was markedly decreased by co-incubation with 100 μM FPP (Figures 4A,C). Incubation of explants with 100 μM FPP alone showed no ototoxic effect. Thus, in the glutamate toxicity model, as in the *in vivo* model, zoledronate restored synaptic number, and its effect on synapses was reversed by the downstream intermediate that it normally blocks, indicating that the drug exerts its effect on synaptogenesis by blocking production of FPP.

## Discussion

We show here that zoledronate has a striking effect on primary afferent synapses of the auditory system, reversing the damage done by noise exposure and leading to a recovery of peripheral function. Hearing loss in cochlear synaptopathy is characterized by irreversible loss of afferent synapses on IHCs that can be assessed by a reduction of the amplitude of wave I of the ABR. Treatment with zoledronate after exposure to noise led to regeneration of IHC synapses and recovery of ABR wave I amplitudes, indicating effective reversal of synaptopathy.

We utilized the CBA/CaJ mouse line because, once established, synaptic loss at the primary afferent synapse is permanent and no spontaneous regeneration occurs. There are currently no available treatments for hearing loss due to cochlear synaptopathy. The irreversibility of this clinical problem can be ascribed to the lack of regeneration of cochlear cells including SGNs (Liberman et al., 2016).

**FIGURE 5:**
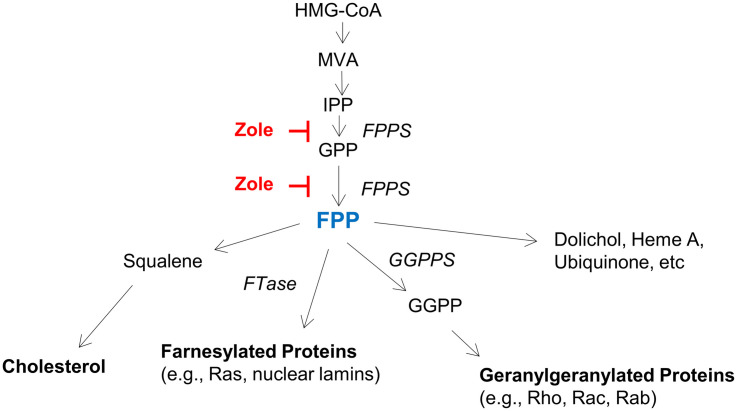
The mevalonate pathway, highlighting key enzymes and intermediates. HMG-CoA (3-Hydroxy-3-methylglutaryl-CoA), MVA (mevalonate), IPP (isopentanyl-pyrophosphate), FPP (farnesyl pyrophosphate), GGPP (geranylgeranyl pyrophosphate), FPPS (farnesyl pyrophosphate synthase), GGPPS (geranylgeranyl pyrophosphate synthase), and FTase (farnesyl transferase).

By demonstrating FPPS expression in both the adult and neonatal cochlea, and using FPP to abolish the regenerative effect of zoledronate on cochlear synapses *in vitro*, we show that the therapeutic effect of zoledronate is due to FPPS inhibition. This is consistent with the known mechanism of action of nitrogen-containing bisphosphonates, which act on the mevalonate–cholesterol pathway by inhibiting FPPS (Figure 5). Supporting a role for the mevalonate pathway, previous work has shown that inhibiting HMG-CoA reductase, which is further upstream in the mevalonate pathway, protected against noise-induced hearing loss in guinea pigs and mice (Park et al., 2012; Richter et al., 2018). Cholesterol metabolism is important in neurodegenerative diseases and strongly associated with SNHL (Malgrange et al., 2015). Inhibitors of the mevalonate pathway are potent neurite-outgrowth-promoting agents (Li et al., 2016).

Farnesyl pyrophosphate synthase inhibition may promote synaptic regeneration via multiple mechanisms. First, zoledronate may prevent prenylation and thereby affect synaptic plasticity and neuroinflammation. Specifically, prenylation involves protein farnesyl transferase catalyzing transfer of farnesyl group from FPP to a range of proteins, including Rho GTPase signaling molecules such as Rac1, Cdc42, and RhoA, which are known to regulate neurite outgrowth and synaptic spine dynamics (Haditsch et al., 2009; Briz et al., 2015). These molecules are critical in the neural circuit formation especially during neurodevelopment (Tolias et al., 2011). Importantly, heterozygous deletion of farnesyl transferase can rescue spatial learning and memory function in a mouse model of Alzheimer’s disease (Cheng et al., 2013).

Second, zoledronate may exert its effect by lowering FPP directly. FPP is an endogenous activator of transient receptor potential cation channel, vanilloid type (TRPV) 3 (Bang et al., 2010), and TRPV3 deficiency protects against kanamycin-induced hearing loss (Wang et al., 2019). While Wang et al. (2019) attribute the protective effect of TRPV3 deficiency to compensatory TRPV4 upregulation, we hypothesize that TRPV3 activation by FPP may play a role in ototoxicity.

Third, zoledronate may lead to upregulation of neurotrophic factors, such as glial cell line-derived neurotrophic factor (GDNF) or insulin growth factor-1 (IGF-1). Different bisphosphonates, including zoledronate, upregulate TGF-beta expression (Jia et al., 2013; Manzano-Moreno et al., 2018), and TGF-β1 can increase GDNF production by upregulating GDNF and furin gene expression (Yin et al., 2020). Moreover, GDNF overexpression in neuronal progenitors leads to downregulation of four key enzymes of the cholesterol-synthesis pathway, including farnesyl-diphosphate synthetase, resulting in decreased production of farnesyl-pyrophosphate (Pahnke et al., 2004). It is relevant that continuous intracochlear GDNF infusion increases survival of SGNs after noise-induced IHC lesions (Keithley et al., 1998; Ylikoski et al., 1998). Etidronate, a non-nitrogen-containing bisphosphonate, can protect retinal ganglion cells via IGF-1 (Su and Huang, 2018), and IGF-1 can regenerate cochlear synapses in the KA *in vitro* model (Yamahara et al., 2019).

Fourth, zoledronate may reduce oxidative stress and apoptosis, analogous to etidronate that rescued spatial memory and upregulated long-term potentiation proteins, such as calcium/calmodulin-dependent protein kinase II (CaMKII), NMDAR 2B, and PSD95 in a model of vascular dementia (Li et al., 2017). The mechanism of that effect was the reduction of oxidative stress and apoptosis, measured by superoxide dismutase (SOD) and malondialdehyde (MDA) activities, and BCL-2 respectively (Li et al., 2017).

In addition, the observation that co-incubation of cochlear explants with zoledronate did not completely abolish its regenerative effect suggests that exogenous FPP partly inhibited FPPS, as FPP is a known allosteric inhibitor of FPPS (Park et al., 2017). Alternatively, zoledronate could be acting through additional, yet to be identified pathways. A similar conclusion was reached when trying to explain neuroprotective effects of statins, which are different inhibitors of the HMG-CoA pathway (Roy et al., 2015). Relative contributions of these various pathways to our observed regenerative effect of zoledronate on cochlear synapses will need to be examined in future studies.

Neurotrophins, which are required for development of the cochlear innervation in the embryo, are effective in the protection of neurons from damage due to toxins or other insults. Previous studies have demonstrated important roles for glial cells in neuronal development, survival, and regeneration. Supporting cells that surround hair cells in the cochlear sensory epithelium secrete brain-derived neurotrophic factor (BDNF) and neurotrophin-3 (NT3) (Hansen et al., 2001). Our results showing predominant immunoreactivity for FPPS in cells surrounding SGNs support an important role of neuron–glia interactions in promoting synaptic regeneration. This interaction may be mediated, at least in part, by GDNF, as explained above, or other neurotrophins. The kainate excitotoxicity results also show that the drug is effective in restoring synapses damaged by glutamate toxicity, which is thought to be the mechanism of noise damage as well (Liberman and Kujawa, 2017). Consistent with our previous description of zoledronate’s direct action on spiral ganglion cells (Schaette and McAlpine, 2011), another study found an effect of a related bisphosphonate, risedronate, *in vitro* (Kempfle et al., 2018). However, that study was focused on the synthesis of a risedronate conjugate with a neurotrophic agent to deliver the agent to cochlear neurons via risedronate’s binding to the surrounding bone. NT3 can regenerate cochlear synapses after acoustic overexposure when delivered locally in mice (Suzuki et al., 2016) and guinea pigs (Sly et al., 2016). NT3 is thought to be more potent than BDNF in these experiments. Application of therapeutic peptides in other neurodegenerative diseases have identified challenges to neurotrophin-based therapies (Weissmiller and Wu, 2012) and small-molecule drugs have obvious advantages over peptides, including potential for systemic delivery. Intracellular signaling elicited by neurotrophins is mediated in part by protein prenylation (Li et al., 2013), suggesting a link between neurotrophins and bisphosphonates. While Kempfle et al. (2018) found that 400 nM risedronate alone had a minor positive effect on the growth of neurites *in vitro*, we found a much more robust effect with 1 μM zoledronate on promoting neurite extension and synapse regeneration *in vitro* and *in vivo*. Differences in the magnitude of the observed effect may reflect differences in drugs’ neuroregenerative potential, drug concentration, model systems, and research foci of the two studies.

The first report of bisphosphonates to treat otologic disease employed the non-nitrogen-containing bisphosphonate etidronate for otosclerosis (Kennedy et al., 1993). This double-blind prospective study showed a non-significant trend toward hearing stabilization and improvement in the etidronate group compared to the placebo group (Kennedy et al., 1993). In a 9-year follow-up study at our institution, patients with cochlear otosclerosis treated with the third-generation bisphosphonate zoledronate showed stabilization or improvement of SNHL and did not exhibit any side effects (Jan et al., 2017). Despite the broad use of bisphosphonates, there are only three reported cases of ototoxicity after etidronate or pamidronate therapy (Reid et al., 1995; Yasil et al., 1998).

The reversal of synaptic loss by bisphosphonates may have implications for other neurodegenerative disorders involving synaptic loss, as well as for an understanding of mechanisms of synaptic modulation important for neural plasticity. The clear effect on synapses helps to solve the mystery of how these drugs, originally established for diseases of bone loss, can exert an effect on neural growth and connectivity. Bisphosphonates can effectively manage complex regional pain syndrome by poorly understood mechanisms (Giusti and Bianchi, 2015). The mevalonate pathway regulates neural growth and survival via prenylation of proteins (Cheng et al., 2013) and is thought to play a role in declining cognitive function in Alzheimer’s disease (Hottman and Li, 2014).

The finding that zoledronate regenerates cochlear synapses may explain previous reports that show that zoledronate improved or stabilized progressive SNHL in patients with otosclerosis. Our results have transformative translational implications for hearing loss and suggest that zoledronate could be repurposed for the treatment of SNHL with cochlear neuropathy and associated compromised ability to understand words in noise, tinnitus, and hyperacusis. There is potentially a long therapeutic window because cell bodies of SGNs can survive for many months (in animal models) and years (in humans) after peripheral synaptic and neurite loss (Liberman and Kujawa, 2017). Future studies will thus need to characterize the latest effective time point of zoledronate administration after synaptopathic noise exposure and the most appropriate dose and mode of administration.

## Materials and Methods

### Animals and Experimental Design

Male CBA/CaJ mice were purchased from Jackson Laboratories in three different replication cohorts. Cochlear function was tested by measuring ABRs and DPOAEs 2–4 days before noise exposure at 7 weeks of age (*N* = 22 mice) or 8 weeks of age (*N* = 3 mice). Animals were then exposed to a noise band known to destroy cochlear synapses and cause cochlear neuropathy. Mice were randomly assigned to a group receiving subcutaneous (SC) injection of 0.5 mg/kg zoledronate (Novartis Pharmaceuticals, AZ) (*N* = 13 animals) or a control group receiving SC injection of vehicle (saline) (*N* = 12 animals) 24, 48, and 72 h after noise exposure. Fourteen days after noise exposure, cochlear function was re-assessed with ABRs and DPOAEs, and the cochleae were fixed by intracardiac perfusion and removed for histological analyses. All experimental procedures were approved by the Institutional Animal Care and Use Committee of Massachusetts Eye and Ear and conducted in accordance with the NIH Guide for the Care and Use of Laboratory Animals.

### Noise Exposure

Mice were exposed to octave-band noise (8–16 kHz) for 2 h at 97 dB sound pressure level (SPL) in a reverberant, acoustically transparent wire box on a rotating platform. Animals were awake and unrestrained during noise exposure. The noise was created digitally using a fifth-order Butterworth filter, amplified through a power amplifier (Crown D75A), and delivered by a loudspeaker (JBL2446H) coupled to an exponential horn in the roof of the box. Exposure levels were measured in each cage with a 0.25-inch Brüel and Kjaer condenser microphone.

### Cochlear Function Testing

Auditory brainstem responses and distortion product otoacoustic emissions were recorded as detailed previously (Jensen et al., 2015; Suzuki et al., 2016). The mice were anesthetized with ketamine (100 mg/kg) and xylazine (10 mg/kg) administered intraperitoneally. A custom acoustic system was used consisting of two miniature earphones serving as sound sources (CDMG150 008-03A, CUI) and a microphone (FG-23329-PO7, Knowles) coupled to a probe tube to measure sound pressure near the eardrum. DPOAEs were measured as ear canal pressure in response to two tones presented into the ear canal (f1 and f2, with f2/f1 = 1.2) at half octave steps, from f2 = 5.66–45.25 kHz, and in 5-dB intensity increments from 15 to 80 dB SPL. ABR responses to 5-ms tone pips were measured between subdermal electrodes (positive behind the ipsilateral pinna, negative at the vertex, and ground at the tail), amplified 10,000 times, and filtered (0.3–3.0 kHz). For each frequency and sound level, 512 responses were recorded and averaged using custom LabVIEW data-acquisition software run on a PXI chassis (National Instruments Corp., Austin, TX, United States). The ABR waveforms were stacked from lowest to highest SPL and visually inspected to define threshold as the first level at which a repeatable wave I was detected. ABR data were acquired in 5-dB intensity increments below threshold and at 5- to 10-dB increments above threshold. When data were acquired in 10-dB increments at some sound levels, the values at 5 dB increments are linear interpolates of the adjacent data points. ABR wave I amplitude was measured peak-to-peak using the ABR Peak Analysis software (Eaton-Peabody Laboratories). Cochlear function testing and data quantification were performed by the researcher blinded to the treatment group.

### Cochlear Whole Mounts and Quantitative Confocal Fluorescence Immunohistochemistry

Deeply anesthetized animals were intracardially perfused with 4% paraformaldehyde (PFA). Both bullae were opened, and the round and oval window membranes were punctured to allow gentle intracochlear perfusion with PFA. Cochleae were extracted and post-fixed for 2 h in 4% PFA and decalcified in 0.12 M EDTA for 72 h. Whole mounts of the organ of Corti were prepared by microdissecting the spiraling cochleae into 6 pieces, blocking them with 5% normal horse serum (NHS) and 0.3% Triton X-100 (TX-100; Integra Chemical, WA, United States) in PBS for 1 h at room temperature, and immunostaining overnight at 37°C with the following primary antibodies diluted in 1% NHS with 0.3% TX-100 rabbit anti-myosin 7A at 1:200 (#25-6790 Proteus Biosciences, CA, United States) to label hair cells, mouse (IgG1) anti-CtBP2 (C-terminal Binding Protein) at 1:200 (#612044, BD Transduction Labs, CA, United States) to label pre-synaptic ribbons, and mouse (IgG2a) anti-GluA2 (Glutamate receptor subunit A2) at 1:2000 (#MAB397, Millipore Sigma, MA, United States) to label post-synaptic receptor patches. After washing in PBS three times, cochlear pieces were incubated twice in species-appropriate secondary antibodies: Alexa Fluor 488-conjugated goat anti-mouse (IgG2a) at 1:1000 (#A21131, Life Technologies, CA, United States), Alexa Fluor 568-conjugated goat anti-mouse (IgG1) at 1:1000 (#A21124, Life Technologies, CA, United States), and Alexa Fluor 647-conjugated chicken anti-rabbit at 1:200 (#A21443, Life Technologies, CA, United States). Specimens were first imaged at low magnification (×10 objective) using a fluorescent microscope (E800, Nikon) and a custom ImageJ plug-in^1^ to create a cochlear frequency map. Cochlear whole mounts were imaged with a confocal microscope (SP8, Leica) in the 32.0-kHz region while focusing on the presynaptic ribbons in the basolateral portion of IHCs. A glycerol-immersion 63× objective (1.3 N.A.) and 2.4× digital zoom were used to acquire image stacks with the *z* dimension sampled at 0.25 μm and the span adjusted to include all synaptic elements in the *xy* field of view. Each *z*-stack included 8–12 adjacent IHCs, and two contiguous *z*-stacks were obtained in each frequency location. *z*-Stacks were transferred to the Amira imaging software (Visage Imaging, version 5.2.2). The connected components and iso-surface functions were used to quantify ribbon numbers, which were expressed as a mean (ribbons/IHC) of the two areas per frequency region. Synaptic quantification was performed by the researcher blinded to the treatment group.

### Fluorescence Immunohistochemistry of Frozen Sections

Decalcified cochleae were cryoprotected in increasing sucrose concentrations (10 and 20% each for 1 h, followed by overnight incubation in 30% sucrose in PBS at 4°C), embedded in Tissue-Tek O.C.T. Compound (Sakura Finetek, CA, United States), and frozen and stored at −80°C before future use. Cochleae were sectioned at 12 μm on a cryostat (CM3050S, Leica Instruments), mounted on glass slides and stored at −20°C. Tissue sections were washed with PBS three times, blocked with 5% NHS and 0.3% TX-100 in PBS for 1 h at room temperature, and immunostained overnight at 37°C with the following primary antibodies diluted in 1% NHS and 0.3% TX-100: rabbit anti-FDPS antibody at 1:1000 (PA5-28228, Thermo Fisher, MA, United States) to label FPPS (also known as FDPS), chicken anti-NF-H at 1:2500 to label neurites (AB5539, Millipore Sigma, MA, United States), and mouse anti-Myo 7A at 1:10 (138-1, Developmental Studies Hybridoma Bank, IA, United States) to label hair cells. Sections were then washed with PBS and incubated with species-appropriate secondary antibodies (Alexa Fluor 488 anti-rabbit; 1:500, Alexa Fluor 568 anti-chicken; 1:500, Alexa Fluor 647 anti-mouse; 1:500, Thermo Fisher Scientific, MA, United States) for 1 h at room temperature. Specimens were imaged with a Leica SP8 confocal microscope. As a negative control, primary antibodies were omitted from the staining protocol. This resulted in no specific signal. As a positive control, immunostaining using unrelated primary antibodies gave rise to different specific patterns of positive cells than what we observed in the current study.

### *In vitro* Model of Cochlear Synaptopathy

Cochlear explant cultures were prepared as previously described by our laboratory (Landegger et al., 2017). Briefly, postnatal day 4 CBA/CaJ wild-type mice (Jackson Laboratory, ME, United States) were decapitated, the temporal bones extracted, and the otic capsule was dissected away from the cochleae in Hank’s Balanced Salt Solution (Life Technologies, CA, United States). The spiral ligament and stria vascularis were gently stripped away from base to apex. The middle part was carefully dissected into a more apical and more basal part, containing sensory hair cells and SGNs. The tectorial and Reissner’s membrane were removed. Explants were left overnight to attach onto 10 mm glass coverslips coated with Cell-Tak (#354241, BD Biosciences, CA, United States) in a 35-mm culture dish with 4 wells in culture medium consisting of 98% DMEM, 1% ampicillin, and 1% N2 supplement at 37°C and 5% CO_2_ levels in sterile conditions. After microscopically confirming attachment, explants were treated with 0.5 mM KA (# ab120100, Abcam, MA, United States) diluted in culture medium to induce glutamatergic excitotoxicity (Wang and Green, 2011; Kempfle et al., 2018; Yamahara et al., 2019). After 2 h, medium was exchanged, and explants were either cultured in culture medium or supplemented with 1 μM zoledronate alone or with mevalonate pathway intermediate FPP for 24 h. FPP ammonium salt (#F6892) was purchased from Sigma-Aldrich. After treatment, cochlear explants were rinsed in PBS, fixed with 4% PFA (Electron Microscopy Sciences, PA, United States) in PBS for 20 min, washed with PBS, and blocked in a blocking buffer consisting of 5% NHS (Sigma-Aldrich, MO, United States) with 1% TX-100 (Integra Chemical, WA, United States) for 1/2 h at room temperature. The following primary antibodies diluted in 1% NHS with 0.3% TX-100 were used for immunostaining and incubated overnight at room temperature: rabbit anti-myosin 7A at 1:500 (#25-6790, Proteus Biosciences, CA, United States) to label hair cells, mouse (IgG1) anti-CtBP2 (C-terminal binding protein) at 1:1000 (#612044, BD Transduction Labs) to label pre-synaptic ribbons, mouse (IgG2a) anti-PSD95 (post-synaptic density 95) at 1:1000 (#75-028, Neuromab, CA, United States) to label post-synaptic neural synapse patches, and chicken NF-H at 1:2500 (AB5539, Millipore Sigma, MA, United States) for neurites. After washing in PBS three times, explants were incubated in species-appropriate secondary antibodies at 1:500 dilution for 11/2 h: Alexa Fluor 647-conjugated goat anti-mouse (IgG2a) (#A21131, Life Technologies, CA, United States), Alexa Fluor 568-conjugated goat anti-mouse (IgG1) (#A21124, Life Technologies, CA, United States), Pacific blue-conjugated chicken anti-rabbit (#A21443, Life Technologies, CA, United States), and Alexa Fluor 488-conjugated goat anti-chicken (#A11039, Life Technologies, CA, United States). After washing three times in PBS, coverslips were mounted on glass slides using Vectashield (#H-1000, Vector Laboratories, CA, United States), and the edges were sealed with clear nail polish (Electron Microscopy Sciences, PA, United States). Specimens were imaged with a Leica SP8 confocal microscope, first at 20× for an overview of the specimen, then focusing separately on standardized areas to the explant’s right and left, images were taken at 63× and with additional 2.4× digital zoom to visualize the entire organ of Corti and the IHC-SGN synapse, respectively. *z*-Stacks were transferred to Amira imaging software (Visage Imaging, version 5.2.2). Connected components and iso-surface functions were used to create 3D renderings to count for synaptic juxtapositions as previously described (Suzuki et al., 2016). The number of IHCs and NF bundles were manually counted per 100 μm. NF bundles were counted and assessed approximately 10 μm beneath IHCs (Dilwali et al., 2015). Quantification was performed by the researcher blinded to the treatment group.

### Statistical Analysis

Statistical analysis was performed using GraphPad Prism 8.2.1. Statistical significance in ABR and DPOAE data (threshold shifts, wave I amplitude) was determined using ordinary two-way ANOVA with subsequent Tukey’s multiple comparisons test. *In vivo* synaptic ribbons and juxtapositions were analyzed with two-tailed *t*-test. For *in vitro* synaptic juxtapositions, Tukey’s multiple comparison test was employed following one-way ANOVA. A probability value of *P* < 0.05 was considered statistically significant. All data are presented as means ± standard errors of the mean (SEMs).

## Data Availability Statement

The datasets generated for this study are available on request to the corresponding author.

## Ethics Statement

The animal study was reviewed and approved by the Institutional Animal Care and Use Committee of Massachusetts Eye and Ear.

## Author Contributions

KS and AE conceived the project, designed the experiments, and jointly supervised all aspects of the research. RS, MT, LL, SV, HH, and SK performed the experiments. All authors analyzed the data. RS, KS, and AE wrote the manuscript, with editorial input from all authors.

## Conflict of Interest

KS and AE are holders of patent US9670288B2 titled “Osteoprotegerin in neuroprotection.” AE is a cofounder and scientific advisor to Decibel Therapeutics and Audion Therapeutics. The remaining authors declare that the research was conducted in the absence of any commercial or financial relationships that could be construed as a potential conflict of interest.

## Footnotes

1 http://www.masseyeandear.org/research/otolaryngology/investigators/laboratories/eaton-peabody-laboratories/epl-histology-resources/
